# Tuberculosis of the Genito-Urinary Organs, Male and Female

**Published:** 1898-04

**Authors:** 


Tuberculosis of the Genito-Urinary Organs, Male and Female. By N. Senn, M. D., Ph. D., LL. D., Professor of Surgery and Clinical Surgery, Rush Medical College; Attending Surgeon to Presbyterian Hospital; Surgeon in Chief, St. Joseph's Hospital, Chicago. Illustrated. W. B. Saunders, 925 Walnut St., Philadelphia. 1897. Cloth, $3.

This work appears to fill a gap in medical literature, as no work of similar character has been published heretofore. Prof. Senn is an epoch-making surgeon, and this work, like all others from his pen, bears the impress of originality and thoroughness on every page. While the work will, no doubt, commend itself especially to those who are confining their practice to genito-urinary diseases, yet, as a fact, it is equally valuable to the general practitioner. After a perusal, we were convinced that many cases treated by us in the past for gonorrhea, syphilis and chancroid were really tuberculosis, and that if they had been treated as such, results would have been better. The subject is taken up in a systematic manner, each anatomical region being separately discussed, and the author has drawn largely upon his own rich clinical experience in laying down the treatment.

The work consists of 300 pages, and is divided into ten chapters. Excellent illustrations add greatly to the finish of the work, which is faultless in mechanical execution. The work is recommended without reserve. J.

